# Supplementary material for: The Acquisition of Multidrug-Resistant Bacteria in Patients Admitted to COVID-19 Intensive Care Units: A Monocentric Retrospective Case Control Study
Source: Microorganisms. 2020 Nov 19;8(11):1821. doi: 10.3390/microorganisms8111821 (PMC7699265; doi:10.3390/microorganisms8111821)
Supplement: Supplementary file 1 [file microorganisms-08-01821-s001.pdf]

Electronic supplemental material

The development of bacterial antimicrobial resistance in critically ill patients admitted to a COVID 19 unit : a monocentric retrospective study.

Elisa G Bogossian , Fabio S Taccone, Antonio Izzi, Nicolas Yin, Alessandra Garuffi , Stephane Hublet[4], Hassan Njimi, Julie Gorham, Baudoin Byl Alexandre Brasseur, Maya Hites, Jean-Louis Vincent, Jacques Creteur, David Grimaldi

Corresponding author:

Dr David Grimaldi, Soins Intensifs, ERASME, Route de Lennik 808, 1070 Bruxelles, Belgium. DG had full access to all the data in the study and had final responsibility for the decision to submit for publication.

E-mail: david.grimaldi@erasme.ulb.ac.be

Orcid number : 0000-0001-8428-065X

Phone: + 32 02 555 4445

Electronic supplemental material

Table S1: Comparison between MDRB + and - patients including COVID-19 and SAH patients, matched on mechanical ventilation and ICU length of stay

	MDRB - (N=104)	MDRB + (N=40)	p-value#
COVID 19 (vs SAH), n (%)	48 (46)	24 (60)	0.19
Age; years, mean (±SD)	56 (±16)	58 (±13)	0.53
Male gender, n (%)	55 (53)	24 (60)	0.46
Charlson comorbidity index, median (IQR)	1 (0-3)	1 (0-3)	0.71
Hospitalization in the previous 6 months, n (%)	12 (12)	2 (39)	0.35
Transfer from other hospitals, n (%)	11 (11)	9 (23)	0.10
Patients MDRB + at admission, n (%)	7 (7)	2 (5)	0.99
SAPS 3 score, median (IQR)	41 (31-54)	47 (36-60)	0.17
SOFA sore, median (IQR)	5 (2-9)	8 (4-10)	0.03
Central venous catheter*, n (%)	76 (73)	36 (90)	0.03
Urinary tract catheter*, n (%)	72 (69)	34 (85)	0.06
Mechanical ventilation*, n (%)	55 (53)	28 (70)	0.09
Length of MV*; days, median (IQR)	1 (0-8)	9 (0-15)	0.005
Vasopressor, n (%)	59 (57)	35 (88)	0.001
Acute kidney injury, n (%)	21 (20)	14 (35)	0.08
Renal replacement therapy, n (%)	11 (11)	7 (18)	0.27
ECMO, n (%)	8 (8)	4 (10)	0.74
Surgery*, n (%)	40 (38)	13 (32.5)	0.57
Antimicrobial therapy* , n (%)	58 (56)	35 (88)	0.001
Length of antimicrobial therapy*; days, median (IQR)	2 (0-5)	6 (4-7)	0.001
Length of exposure; days, median (IQR)	6 (3-20)	13 (9-20)	0.003
ICU LOS; days , median (IQR)	6(3-20)	27 (21-34)	<0.001
ICU LOS of survivors; days, median (IQR)	4 (2-21)	28 (25-34)	<0.001
Hospital LOS; days, median (IQR)	19(11-33)	38(24-62)	<0.001
Hospital LOS of survivors; days, median (IQR)	23 (12-39)	52 (33-64)	<0.001
ICU mortality, n (%)	28 (27)	10 (25)	0.99
Hospital mortality, n (%)	31 (30)	10 (25)	0.68

*Variables collected before MDRB acquisition.
#p value was obtained using Qui square or Fisher exact test, T- Student or Mann-Whitney test as appropriate.
COVID-19: Coronavirus disease 19; MDRB: multidrug resistant bacteria; SAPS 3: Simplified acute physiology score 3; SOFA: Sequential organ failure assessment ; MV: mechanical ventilation; LOS: length of stay

Electronic supplemental material

Figure S1: Distribution of MDRB bacteria acquired during ICU according to mechanism of resistance in SAH patients

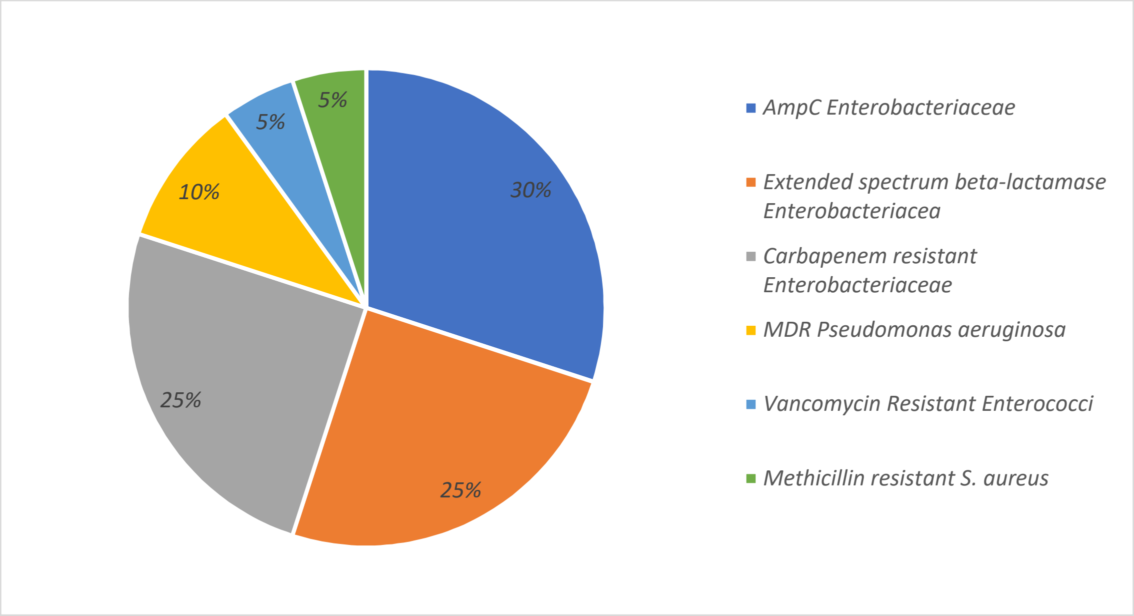

AmpC beta- lactamase (AmpC) *Enterobacteriaceae* (N=6):
Morganella morganii (N=1); *Enterobacter aerogenes* (N=2); *E. cloacae* (N=3)

Extended spectrum -lactamase (ESBL) *Enterobacteriaceae* (N=5):
Klebsiella oxytoca (N=1), *Escherichia coli* (N=3), *K. pneumoniae* (N=1)

Carbapenem resistant *Enterobacteriaceae* (CRE) (N=5):
E. cloacae (N=1), *K. pneumoniae* (N=3), *E. coli* (N=1),

Vancomycin resistant enterococci (VRE) N=1:
Enterococcus faecium (N=1)

Methicillin resistant *Staphylococcus aureus* (MRSA) (N=1)

Electronic supplemental material

Table S2: Type of antibiotics used in COVID- 19 patients (N=56/72).

	All patients (N=56)	MDRB - (N=34)	MDRB + (N=22)
Amoxicillin – clavulanic acid, n (%)	13 (23)	8 (24)	5 (23)
Cephalosporin 1st and 2nd generations, n (%)	1 (2)	1 (3)	0
Cephalosporin 3rd and 4th generation, n (%)	10 (18)	7 (21)	3 (14)
Piperacillin -tazobactam, n (%)	24 (43)	12 (35)	12 (55)
Vancomycin, n (%)	2 (4)	2 (6)	0
Carbapenems, n (%)	6 (11)	4 (12)	2 (9)

Overall p-value=0.62 There were no statistically significant differences between columns as calculated by the Bonferroni method.

Table S3: Characteristic of the septic shock cohort

	Septic shock patients (N=60)
Age, median (IQR)	63 (51-72)
Gender, n (%)	40 (67)
Charlson Comorbidity Index, median (IQR)	5 (3-6)
SOFA score, n (%)	11 (8-13)
MV, n (%)	41 (68)
Vasopressors, n (%)	59 (98)
ECMO, n (%)	8 (13)
RRT, n (%)	18 (30)
Previous surgery, n (%)	21 (35)
Previous hospitalization, n (%)	30 (50)
MV before MDR, n (%)	38 (63)
Central venous catheter, n (%)	59 (98)
Antibiotic before MDR, n (%)	59 (98)
Time at risk, median (IQR)	6 (3-12)
ICU LOS, median (IQR)	8 (3-20)
Hospital LOS, median (IQR)	19 (12-41)
ICU mortality, n (%)	21 (35)
Hospital mortality, n (%)	21 (35)

LOS: Length of stay; IQR: interquartile range; SOFA: sequential organ failure assessment; MV: mechanical ventilation; ECMO: extra corporeal membrane oxygenation; RRT: renal replacement therapy; MDR: multidrug resistance; LOS: Length of stay. We identified 203 patients admitted to our ICU from 2016-2019 due to septic shock. Of these we were able to match 60 patients to the patients admitted to the COVID- 19 units based on the use of mechanical ventilation and ICU length of stay. This table shows the characteristics of the selected 60 patients.

Electronic supplemental material

Figure S2: MDRB acquisition rate from 2016 to 2019 in the SAH and septic shock cohorts

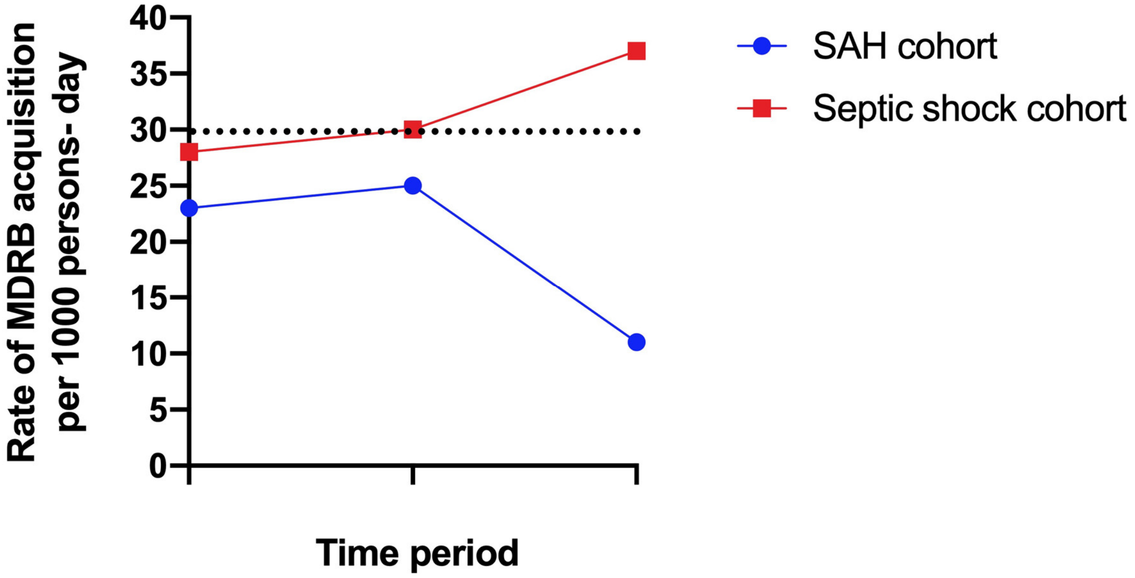

MDRB: multidrug resistant bacteria; SAH: subarachnoid hemorrhage
The dotted line represents the rate of MDRB acquisition in patients admitted to COVID-19 units. The cohorts were divided according to tertile starting from the left to the right: earliest tertile (2016-2017), middle tertile (2017-2018) and latest tertile (2018-2019).
